# Effect of COVID-19 on Brazilian cesarean and prematurity rates: a cross-sectional study

**DOI:** 10.61622/rbgo/2025rbgo6

**Published:** 2025-03-17

**Authors:** Clarissa Suzart, José Paulo de Siqueira Guida

**Affiliations:** 1 Universidade Estadual de Campinas Campinas SP Brazil Universidade Estadual de Campinas, Campinas, SP, Brazil.

**Keywords:** Cesarean delivery, Prematurity, Maternal mortality, Infant, newborn, Live birth, Pandemics, COVID-19

## Abstract

**Objective:**

To investigate the relationship between prematurity and cesarean section rate in Brazil during the beginning of COVID-19 pandemic.

**Methods:**

Utilizing the Robson Classification, this study analyzed data from the Brazilian Ministry of Health's Live Births Panel, comparing CSR) and group 10 (preterm deliveries) between 2019 (pre-pandemic) and 2021 (pandemic) in each of Brazilian states and the overall country. The prematurity and CSR were compared using prevalence ratio and confidence interval, and p-value was obtained. The variation of prematurity and CSR were compared through the coefficient of determination (R2).

**Results:**

A total of 5,522,910 deliveries were evaluated during the period. The CSR increased from 56.34% to 57.05% (p<0.01), and the frequency of preterm deliveries rose from 8.99% to 9.13% (p<0.01). The CSR increased in 23 States and decreased in 4 States, while the prematurity rate increased in 16 States and decreased in 10 States. A positive relationship between the increase of CSR and prematurity was observed during COVID-19, with an R2 value of 0.3121, suggesting a moderate association between these two variables.

**Conclusion:**

Between 2019 (pre-COVID-19 pandemic) and 2021 (the first full year of the COVID-19 pandemic), there was an increase in prematurity and CSR in Brazil. These increases were observed in most Brazilian states and may be correlated. However, it is impossible to establish a cause-effect relationship given the design of this study.

## Introduction

Since 1985, the World Health Organization (WHO) has stated that cesarean rates exceeding 10% to 15% are not associated with improvements in maternal mortality rates.^(1)^ Despite this, global cesarean rates have doubled over the past 15 years, reaching 21% of all births and growing by about 4% annually.^(2)^ Brazil ranks second in performing cesareans, surpassed only by the Dominican Republic.^(3)^ This trend is increasingly concerning because, although cesarean sections are life-saving surgeries and represent significant advancements in obstetric care, their unnecessary performance is linked to short- and long-term adverse outcomes for both women and newborns.

The COVID-19 pandemic has exacerbated these concerns by significantly impacting healthcare in Brazil, accelerating trends, and exposing systemic vulnerabilities. During the pandemic, reductions in prenatal care led to an increase in severe obstetric complications, often resulting in more cesarean sections as a precautionary measure to avoid unfavorable maternal-fetal outcomes. Additionally, fewer women participated in prenatal education programs, possibly contributing to a lack of informed decision-making regarding childbirth.^(4)^

To address the rise in cesarean rates and promote safer childbirth practices, the WHO has, since 2015, recommended the use of the Robson Classification (RC) to evaluate and monitor cesarean sections across different healthcare settings.^(5,6)^ The RC is a simple, prospective tool that classifies pregnant women into ten mutually exclusive and fully inclusive groups based on six essential obstetric characteristics, such as parity, previous cesarean history, mode of labor onset, gestational age, fetal presentation, and number of fetuses.^(7)^ This system allows for real-time classification and facilitates a deeper analysis of cesarean trends in varying contexts.

In Brazil, the prevalence of preterm deliveries is approximately 12.3%, with regional variations ranging from 14.7% in the Northeast to 11.1% in the Southeast.^(8)^ Among all preterm births, 35.4% were medically indicated, primarily due to conditions such as hypertension and diabetes.^(9,10)^ The remaining 64.6% were spontaneous, with smoking and urinary tract infections identified as significant contributing factors.^(8)^

In this article, we aim to explore the variations in cesarean and prematurity rates in Brazil between 2019 (pre-pandemic) and 2021 (pandemic), analyze the differences across Brazilian states, and examine possible interactions between these two rates. By understanding these dynamics, we can better address the challenges posed by increasing cesarean sections and their potential impact on maternal and neonatal health.

## Methods

We performed a cross-sectional study based on publicly available data provided by the Brazilian Ministry of Health. For this study, the data were extracted from the Live Births Information System of the Brazilian Ministry of Health, an open online database accessible through the Internet, which, in turn, constituted a convenience sample size, virtually including all deliveries in Brazil. All deliveries in Brazil must be notified to the Ministry of Health by the facility where the birth occurred. The Ministry of Health groups this data, anonymizes it, and publishes it through the Live Births Information System.

For this study, the total number of cesarean and vaginal births from the year 2019 (pre-pandemic) and 2021 (pandemic) were extracted and categorized into each of the RC groups. Deliveries not classified into any of the Robson groups (which represented a residual fraction of 2.56% of the entire sample) were excluded from this analysis. The cesarean percentage in each group for each period was calculated and compared using the prevalence ratio (PR), confidence interval (CI), and p-value, which were considered significant if they were less than 0.05.

Subsequently, we obtained the cesarean rate and frequency of group 10 for each of the 27 Brazilian States in 2019 and 2021. We calculated the variation between the two periods and estimated the PR, CI, and p-value. Group 10, which includes women who had preterm births in cephalic presentations, was considered a proxy for maternal severity – because part of preterm deliveries are due to worsening of maternal clinical conditions, and the increase of the rate in the pandemic period could be a consequence of the COVID-19 infection.

The variations in cesarean rates and the frequency of group 10 in both periods for each state were associated through the coefficient determination (also R-squared or R2). This statistical measure determines, in a regression model, the proportion of variance that the interaction between two variables can explain. The result of R2 ranges from 0 to 1, with 0 indicating no explanatory power and 1 indicating perfect explanatory power. This statistical test was not built to imply causation but correlation between the studied variables. The results of R2 are presented in form of graphs, with the line showing the sense of the association (positive or negative) and the value of R2. The statistical analysis was performed using EpiInfo 7.2, and the graphs were built using Microsoft Excel based on the data analyzed by the statistical software.

This study did not undergo ethical evaluation because the data were extracted from the Live Births Information System, a public database from the Brazilian Ministry of Health. This database is available online for access by any citizen. Thus, as these are already anonymized public data, ethical evaluation by the Research Ethics Committee is waived, according to Resolution 466/2012 from the Brazilian government's National Council of Ethics in Research. We followed the STROBE Statement in this article.

## Results

A total of 2,847,293 births occurred in 2019, and 2,675,617 births occurred in 2021. They were evaluated, resulting in a total sample of 5,522,910 deliveries. The cesarean section rate during 2019 was 56.34%, which increased to 57.05% in 2021 (PR 1.0125, CI 1.0110 – 1.0140, p<0.01). The evaluation by Robson groups is presented in table 1, which shows significant variations in cesarean rates across all groups except for groups 6 and 9. Table 1 highlights that the most significant increase in cesarean rates occurred in group 10 (from 52.63% to 54.26%, PR 1.031, CI 1.025 – 1.0364, p<0.01), while the most substantial decrease was observed in group 1 (from 44.02% to 43.76%, PR 0.9942, CI 0.9897 – 0.9987, p=0.0114).

**Table 1:** Comparison of cesarean section rates in Brazil before COVID-19 pandemic (2019) and during the pandemic (2021), according to the Robson Ten Group Classification System

Group of Robson	Cesarean section rate (2019)	Cesarean section rate (2021)	Prevalence ratio	Lowest confidence interval	Highest confidence interval	p-value
1	44.02%	43.76%	0.9942	0.9897	0.9987	0.0114
2	71.71%	72.92%	1.0169	1.0140	1.0198	<0.0001
3	18.61%	19.07%	1.0249	1.0170	1.033	<0.0001
4	48.44%	50.65%	1.0456	1.0397	1.0516	<0.0001
5	85.23%	85.52%	1.0034	1.002	1.0048	<0.0001
6	91.94%	91.72%	0.9976	0.9933	1.002	0.2872
7	88.53%	88.93%	1.0046	1.0003	1.0089	0.0382
8	84.53%	85.69%	1.0138	1.009	1.0186	<0.0001
9	97.42%	97.13%	0.997	0.9906	1.0034	0.3843
10	52.63%	54.26%	1.031	1.0257	1.0364	<0.0001
Total	56.34%	57.05%	1.0125	1.0110	1.0140	<0.0001

The overall variation in the cesarean rate in Brazil during the assessed period was +0.71%. There was a positive variation in this rate in 23 States and a negative variation in 4 others. The variation was statistically significant in 16 states. with the most substantial negative variation occurring in the state of Amapá (-7.40%. PR 0.819. CI 0.755 – 0.889. p<0.001) and the most significant positive variation in Mato Grosso (+5.68%. PR 1.123. CI 1.081 – 1.117. p<0.01). The variation in the cesarean rate for each state is presented in table 2.

**Table 2:** Cesarean section rates in each Brazilian State before COVID-19 pandemic (2019) and during the pandemic (2021)

State	CSR* (2019)	CSR* (2021)	Variation	PR*	Confidence Interval	p-value
Acre	37.34%	39.94%	2.60%	1.059	(0.987 – 1.136)	0.122
Alagoas	46.86%	48.59%	1.73%	1.032	(0.993 – 1.074)	0.115
Amapá	45.08%	37.68%	-7.40%	0.819	(0.755 – 0.889)	<0.001
Amazonas	39.26%	40.74%	1.48%	1.032	(0.999 – 1.064)	0.056
Bahia	41.88%	43.01%	1.13%	1.025	(1.001 – 1.049)	0.039
Ceará	54.19%	57.65%	3.46%	1.074	(1.046 – 1.102)	<0.001
Distrito Federal	55.66%	58.35%	2.69%	1.061	(1.009 – 1.115)	0.021
Espírito Santo	56.80%	58.72%	1.92%	1.039	(0.996 – 1.086)	0.078
Goiás	59.15%	60.31%	1.16%	1.026	0.992 – 1.060)	0.137
Maranhão	43.38%	45.42%	2.04%	1.044	(1.014 – 1.074)	0.004
Mato Grosso	58.79%	64.47%	5.68%	1.123	(1.081 – 1.117)	<0.01
Mato Grosso do Sul	55.11%	56.50%	1.38%	1.029	(0.986 – 1.073)	0.201
Minas Gerais	54.24%	54.49%	0.26%	1.005	(0.986 – 1.025)	0.596
Pará	42.51%	46.97%	4.46%	1.093	(1.068 – 1.119)	<0.001
Paraíba	53.34%	54.82%	1.47%	1.030	(0.990 – 1.072)	0.147
Paraná	59.93%	61.94%	2.00%	1.043	(1.017 – 1.071)	0.001
Pernambuco	44.78%	47.94%	3.16%	1.066	(1.039 – 1.094)	<0.001
Piauí	50.76%	53.18%	2.43%	1.050	(1.005 – 1.097)	0.030
Rio de Janeiro	60.12%	61.55%	1.43%	1.031	(1.009 – 1.054)	0.006
Rio Grande do Norte	57.72%	60.88%	3.15%	1.067	(1.024 – 1.113)	0.002
Rio Grande do Sul	60.97%	62.89%	1.92%	1.043	(1.015 – 1.072)	0.002
Rondônia	59.91%	60.51%	0.60%	1.012	(0.954 – 1.073)	0.713
Roraima	33.73%	30.08%	-3.65%	0.924	(0.865 – 0.986)	0.018
Santa Catarina	55.58%	55.31%	-0.27%	1.033	(1.001 – 1.066)	0.044
São Paulo	55.87%	57.01%	1.13%	1.024	(1.011 – 1.038)	<0.001
Sergipe	45.06%	42.34%	-2.72%	0.945	(0.892 – 1.001)	0.057
Tocantins	53.60%	53.81%	0.21%	1.004	(0.947 – 1.065)	0.913

* Footnote;

CSR - Cesarean Section Rate; PR - Prevalence Ratio

Regarding the size of group 10. which comprises preterm deliveries and serves as a proxy for maternal severity. an increase of 0.15% was observed in the total births occurring before 37 weeks in Brazil over the two studied periods (PR 1.009. CI 1.006 – 1.012. p<0.001). The variation was significant in 16 states. with the most significant increase in the frequency of births classified in group 10 occurring in the State of Roraima (+2.58%. PR 1.117. CI 1.081 – 1.154. p<0.001) and the most substantial decrease in Amapá (-8.58%. PR 0.681. CI 0.654 – 0.709. p<0.001). The frequency of group 10 in each state in both periods and its variation is presented in table 3.

**Table 3:** Frequency of group 10 in each Brazilian State before COVID-19 pandemic (2019) and during the pandemic (2021)

State	G10* (2019)	G10* (2021)	Variation	PR*	Confidence interval	p-value
Acre	11.33%	10.70%	-0.63%	0.967	(0.933 – 1.003)	0.074
Alagoas	7.89%	9.42%	1.54%	1.099	(1.076 – 1.122)	<0.001
Amapá	19.52%	10.94%	-8.58%	0.681	(0.654 – 0.709)	<0.001
Amazonas	10.24%	10.19%	-0.05%	0.997	(0.981 – 1.014)	0.757
Bahia	8.76%	8.31%	-0.45%	0.970	(0.959 – 0.982)	<0.001
Ceará	9.49%	9.95%	0.47%	1.084	(1.069 – 1.098)	<0.001
Distrito Federal	9.37%	9.05%	-0.32%	0.979	(0.955 – 1.005)	0.119
Espírito Santo	7.55%	8.06%	0.51%	1.036	(1.014 – 1.059)	0.002
Goiás	8.79%	8.48%	-0.32%	0.979	(0.963 – 0.996)	0.014
Maranhão	9.12%	8.84%	-0.28%	0.983	(0.968 – 0.997)	0.022
Mato Grosso	8.74%	9.78%	1.04%	1.063	(1.043 – 1.083)	<0.001
Mato Grosso do Sul	9.96%	10.31%	0.35%	1.020	(0.997 – 1.042)	0.091
Minas Gerais	8.79%	8.80%	0.01%	1.001	(0.991 – 1.011)	0.885
Pará	10.19%	10.45%	0.27%	1.014	(1.022 – 1.027)	0.021
Paraíba	8.61%	8.80%	0.19%	1.012	(0.991 – 1.033)	0.257
Paraná	8.18%	8.72%	0.54%	1.037	(1.023 – 1.050)	<0.001
Pernambuco	8.70%	9.09%	0.39%	1.025	(1.011 – 1.039)	<0.001
Piauí	8.56%	8.78%	0.22%	1.008	(0.985 – 1.031)	0.520
Rio de Janeiro	8.72%	9.08%	0.36%	1.023	(1.012 – 1.035)	<0.001
Rio Grande do Norte	10.44%	10.78%	0.34%	1.018	(0.996 – 1.040)	0.107
Rio Grande do Sul	9.41%	9.46%	0.04%	1.003	(0.989 – 1.016)	0.710
Rondônia	7.60%	8.83%	1.23%	1.085	(1.053 – 1.118)	<0.001
Roraima	12.00%	14.59%	2.58%	1.117	(1.081 – 1.154)	<0.001
Santa Catarina	7.98%	8.23%	0.24%	1.017	(1.000 – 1.033)	0.048
São Paulo	8.86%	8.99%	0.13%	1.008	(1.002 – 1.015)	0.016
Sergipe	7.74%	7.83%	0.09%	1.006	(0.977 – 1.036)	0.700
Tocantins	9.08%	9.34%	0.26%	1.015	(0.985 – 1.047)	0.339
Total	8.99%	9.13%	0.15%	1.009	(1.006 – 1.012)	<0.001

* Footnote:

G10: Group 10. according to the Robson Ten Group Classification; PR - Prevalence Ratio

**Figure 1:**
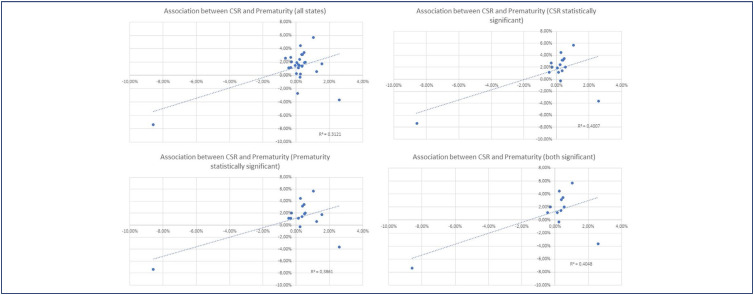
Association between cesarean section rate and prematurity rates in Brazilian States

The variation in cesarean rates and the frequency of preterm delivery (group 10) due to the pandemic showed a direct correlation. indicating that states with a higher increase in prematurity frequency also experienced a higher cesarean rate and vice versa. The R2 value was 0.3121. demonstrating a moderate association between the two variables (Figure 1A). The same association was observed when considering only States where the cesarean rate variation was statistically significant (R2 = 0.4007) and when considering only those where the group 10 frequency variation was statistically significant (R2 = 0.3861) (Figure 1B and 1C). When only states with statistically significant variations in both factors were selected. the same effect continued to be observed (R2 = 0.4048) (Figure 1D).

## Discussion

There was a significant increase in premature birth rates and cesarean rates in Brazil when comparing the year immediately before the onset of the COVID-19 pandemic to the year 2021. There was a direct association between the increase in premature birth frequency and the rise in the cesarean rate.

It is important to consider that this study only evaluated two years (2019 and 2021) because we aimed to understand the impact of the acute event of the COVID-19 pandemic on cesarean and prematurity rates. With our results. we cannot evaluate if the increase in cesarean section rates was already occurring in the years before 2019. From 1994 to 2019. Brazil experienced an increase in cesarean section rates of 2.1%. However. the rate stabilized from 2012 to 2019.^(11)^ Our results suggest that the COVID-19 pandemic impacted cesarean section rates when observing the results from the two included years.

Despite the shortcomings of Brazil's healthcare system being highlighted during this period. we cannot solely attribute the high rates of this procedure to the pandemic. Known determinants include underfunding of obstetric care. a scarcity of labor analgesia in many maternity hospitals in the country. undervaluation of multi-professional work in childbirth assistance. and inappropriate indications for terminating pregnancies due to suspected fetal distress.^(4)^ Thus. an already weakened system further exposes its deficiencies when affected by an unexpected event. such as the beginning of the COVID-19 pandemic.

It is interesting to note that the impact of COVID-19 varied across different countries. A study from Colombia reported that. in that country. the COVID-19 pandemic led to an increase in mean birth weight. a decline in miscarriage risk. and fewer prenatal care visits. They also observed a reduction in preterm deliveries and did not observe changes in cesarean section rates.^(12)^ According to the authors of that study. some limitations of the results are related to the quality of the data when analyzing national databases without accessing raw data – a limitation that also applies to our study.

The classification in group 10 of the Robson Classification does not consider whether a woman had a previous cesarean section. making it impossible to determine how many women underwent their first cesarean. Nonetheless. the high rates of cesarean delivery in this group create a vicious cycle. as induction possibilities and even childbirth management become more limited in women with prior uterine scars. Avoiding the first cesarean. independently of Robson's group the woman is classified in. is one strategy to contain the rise of cesarean rates in the country.^(4,13)^

In Group 10. it is impossible to differentiate between women who experienced spontaneous preterm labor and those for whom preterm delivery was medically indicated. The increase in the size of Group 10 was likely primarily due to a higher number of therapeutic preterm deliveries resulting from maternal or fetal clinical deterioration due to COVID-19 infection. A Brazilian study demonstrated that. during the pandemic. Group 10 increased from 9.2% to 10.4% in a reference hospital.^(14)^ Given that our data are based on the Robson Classification. we cannot assess whether there were attempts at labor induction or if cesarean sections were performed on women already in labor. which presents a limitation. However. our findings reinforce that. on a national level. there was an almost 2% increase in cesarean rates at the onset of the COVID-19 pandemic.

Although studies and major societies like ACOG. RCOG. and ISUOG recommend that the mode of delivery should not be influenced solely by the presence of COVID-19 unless the woman's respiratory condition necessitates urgent intervention for childbirth. a systematic review of articles published between December 2019 and April 29. 2020. showed that 68.9% of patients with COVID-19 underwent cesarean section. with only the disease status being a common indication.^(15)^

In Brazil. laws ensure that women have an active role in deciding the mode of delivery. which is seen as an expression of their autonomy—even when this may be associated with worse maternal and perinatal outcomes. The main reasons Brazilian women request a cesarean section in the absence of medical indications include fear of labor pain. previous traumatic childbirth experiences. and a sense of safety and control over the delivery process.^(16)^ These last two factors may have played a decisive role in the decision to undergo a cesarean section during the COVID-19 pandemic. especially in its early stages. when knowledge about the disease was still limited.

It is known that cesarean sections are associated with short and long-term maternal risks. such as increased chance of death and increased risks of maternal infection and sepsis.^(17)^ However. cesarean sections are life-saving interventions when there is a maternal or fetal indication. preserving outcomes for both.^1^ At the onset of the pandemic. there were concerns about whether the delivery route could impact vertical transmission of the infection and whether COVID-19 infection would necessitate pregnancy termination due to fears of maternal clinical worsening. During 2021. there were recommendations that only the COVID-19 infection should not indicate cesarean as the mode of delivery; however. our study reported a consistent increase in cesarean section rates in the majority of Brazilian states.

A 2020 cohort study involving 18 countries. including Brazil. published in JAMA. showed that women diagnosed with COVID-19 had a lower rate of spontaneous labor and a higher cesarean rate. reflecting higher pregnancy complications in this group. supporting the results of this article. Furthermore. this multinational study showed that most premature births in women with COVID-19 were associated with other clinical indications such as preeclampsia. eclampsia. HELLP syndrome. fetal growth restriction. and fetal distress.^(18)^ The association between COVID-19 and hypertensive disorders in pregnancy remains controversial. A multicenter Brazilian study could not determine this association after evaluating COVID-19 cases in various country regions.^(19)^ However. this possible association may have also justified increased cesarean and premature birth rates in Brazil. as the combination of two severe clinical conditions. COVID-19 respiratory infection. and preeclampsia. might have been decisive for the early resolution of pregnancy through cesarean section.

Our results showed a reduction in the cesarean rate in group 1. which was considered low risk for cesarean birth. due to the characteristics of the women included (nulliparous. single gestation. term. and cephalic presentation). The decrease in cesarean rates in group 1 is an obstetric quality indicator defined by the Joint Commission National Quality Measures. A study performed in 463 hospitals in the United States reported that there was an increase in primary cesareans. However. the study did not specifically apply Robson's Classification in their analysis. That study also showed a significant increase in maternal deaths.^(20)^

On the other hand. there was an increase in cesarean section rates in group 3 – a group in which the expected cesarean section rate must be very low. It is unlikely that these changes were due to the COVID-19 pandemic. and an evaluation of public policies implemented before 2020 is necessary to evaluate its impact on cesarean section rates. according to Robson's groups.

Some state results warrant special attention and should be discussed separately. One such state is Amapá. which significantly reduced cesarean and prematurity rates (−7.40% and −8.58%). This more pronounced reduction than in other evaluated states reinforced the association between the two variations. The decline in both rates may be genuine in that state or due to inadequate data provision to the considered databases. which are filled from data collected in Live Birth Declarations. However. the study design does not allow for assessing the quality of data each state provides to the national database.

The use of public data. already anonymized by the Brazilian Ministry of Health. is a limitation of this study since verifying the raw databases of included data or a significant fraction of it was impossible. Another limitation is that a percentage of cases was excluded because they were not classified into the ten groups. However. this represents a residual fraction of just over 2% of the total considered births. which would have little chance of altering the presented results. On the other hand. this study included a large number of births occurring in Brazil. one of the countries most severely affected by COVID-19. Additionally. the included deliveries represent various Brazilian regions and encompass women from diverse ethnicities and age groups. In this sense. we can consider that the findings of this study are representative of the Brazilian population. However. they should be interpreted cautiously since this study does not allow causation between the increase of cesarean section and prematurity rate but showed an important relationship between them.

## Conclusion

In summary. cesarean and premature birth rates in Brazil significantly increased in 2021 compared to 2019. There was a direct association between the rise in these rates across different Brazilian states. However. it is impossible to define a direct cause-consequence effect between both phenomena.
